# Evaluation of the implementation of a whole-workplace walking programme using the RE-AIM framework

**DOI:** 10.1186/s12889-017-4376-7

**Published:** 2017-05-18

**Authors:** Emma J. Adams, Anna E. Chalkley, Dale W. Esliger, Lauren B. Sherar

**Affiliations:** National Centre for Sport and Exercise Medicine, School of Sport, Exercise and Health Sciences, Loughborough University, Loughborough, UK

**Keywords:** Workplace, Walking, Physical activity, Transport, Commuting, Evaluation

## Abstract

**Background:**

Promoting walking for the journey to/from work and during the working day is one potential approach to increase physical activity in adults. Walking Works was a practice-led, whole-workplace walking programme delivered by employees (walking champions). This study aimed to evaluate the implementation of Walking Works using the RE-AIM framework and provide recommendations for future delivery of whole-workplace walking programmes.

**Methods:**

Two cross sectional surveys were conducted; 1544 (28%) employees completed the baseline survey and 918 employees (21%) completed the follow-up survey. Effectiveness was assessed using baseline and follow-up data; reach, implementation and maintenance were assessed using follow-up data only. For categorical data, Chi square tests were conducted to assess differences between surveys or groups. Continuous data were analysed to test for significant differences using a Mann-Whitney U test. Telephone interviews were conducted with the lead organisation co-ordinator, eight walking champions and three business representatives at follow-up. Interviews were transcribed verbatim and analysed to identify key themes related to adoption, implementation and maintenance.

**Results:**

*Adoption*: Five workplaces participated in Walking Works. *Reach*: 480 (52.3%) employees were aware of activities and 221 (24.1%) participated. *Implementation*: A variety of walking activities were delivered. Some programme components were not delivered as planned which was partly due to barriers in using walking champions to deliver activities. These included the walking champions’ capacity, skills, support needs, ability to engage senior management, and the number and type of activities they could deliver. Other barriers included lack of management support, difficulties communicating information about activities and challenges embedding the programme into normal business activities. *Effectiveness*: No significant changes in walking to/from work or walking during the working day were observed. *Maintenance*: Plans to continue activities were mainly dependent on identifying continued funding.

**Conclusions:**

RE-AIM provided a useful framework for evaluating Walking Works. No changes in walking behaviour were observed. This may have been due to barriers in using walking champions to deliver activities, programme components not being delivered as intended, the types of activities delivered, or lack of awareness and participation by employees. Recommendations are provided for researchers and practitioners implementing future whole-workplace walking programmes.

**Electronic supplementary material:**

The online version of this article (doi:10.1186/s12889-017-4376-7) contains supplementary material, which is available to authorized users.

## Background

A high proportion of the adult population in England do not participate in sufficient physical activity to benefit their health [1]. Increasing population levels of physical activity to improve health and reduce the prevalence and burden of chronic disease is a target for Government policy [2]. In order to support this policy, there is an urgent need to identify strategies which can be implemented at scale in a real world setting, have a wide reach and are effective in increasing and maintaining population physical activity levels.

The workplace is a setting in which there is potential to reach a large number of adults with interventions to promote physical activity and improve health [3, 4]. Almost three quarters (74.6%) of the adult population in England are in employment [5]. A high proportion of employees have sedentary occupations and thus spend long periods of time sitting at work [6, 7]. In addition, 63% to 67% of adults in England travel to work by car [8, 9] and travel to the same workplace every day (72.3%) [8]. Using the workplace to deliver interventions which encourage physical activity, either as part of the journey to and from work or during the working day, may therefore offer potential for increasing physical activity levels.

Walking has been described as the perfect exercise for most adults as it requires no special skills or equipment [10]. It can be undertaken for transport purposes (i.e. to travel from one place to another either alone or in combination with another mode of transport), for recreational purposes or for incidental purposes (e.g. climbing stairs) and it can be carried out in different settings, such as the workplace. Research has shown that workplace interventions can be effective in increasing walking [11–13]. There is also growing evidence that active travel interventions which promote walking to work or aim to encourage a shift from car use to active travel (walking or cycling) can be effective [14, 15]. Promoting walking during the working day (such as encouraging stair use and walking during breaks) and walking as part of the journey to and from work have therefore been recommended as potential strategies to increase physical activity levels [16].

Many of the intervention studies included in the reviews above have been researcher-led and conducted with small numbers of participants in controlled environments. However, in order to have an impact at the population level there is a need for interventions to be delivered in real world settings and embedded into practice. Numerous practice-led interventions have been delivered for which there has been limited reporting of evaluation findings in the scientific literature, though often these types of interventions are not evaluated, or the quality of evaluation is poor with regards to demonstrating effectiveness or assessing implementation and the potential for the intervention to be scaled up [17]. More robust evaluation of practice-led, real-world interventions and reporting in the scientific literature is therefore needed to identify effective interventions and the processes needed for implementation and successful scale-up [18].

Evaluating practice-led interventions being delivered in real-world environments can be challenging. The RE-AIM framework (http://www.re-aim.org) provides a useful model for estimating the potential public health impact of interventions [19] and for assessing the potential for scaling-up interventions [18]. The RE-AIM model includes five dimensions: *Reach* (an individual measure of participation and participant characteristics along with an assessment of representativeness of participants compared to non-participants); *Effectiveness* (individual measures of the positive and negative consequences of the programme including behavioural, quality of life and participant satisfaction outcomes); *Adoption* (organisational measures of the proportion and representativeness of settings that adopt the programme and barriers to adoption); *Implementation* (organisational measures of the extent to which the programme is delivered as intended; individual measures of participant adherence); and *Maintenance* (assessment of long term maintenance of change at the individual level (sustained change in behaviour) and at the organisational level (the extent to which the programme becomes routine/embedded in the everyday culture and norms of an organisation)) [19].

Walking Works was a practice-led, whole-workplace programme which aimed to increase walking to and from work and during the working day. Five workplaces in England participated and employees were recruited to become ‘walking champions’ to help plan and implement the programme. A variety of walking activities were delivered in which all employees were eligible to participate. The aim of this study was to evaluate the Walking Works programme. The objectives were to: 1) Use the RE-AIM framework to evaluate the implementation of the programme at the individual (i.e. employee) and organisational (i.e. workplace) level, and 2) outline the implications of the findings and provide recommendations for future whole-workplace walking programmes which use employees to plan and deliver activities.

## Methods

### Walking Works Programme

Walking Works aimed to encourage people to walk more for all, or some, of their journey to work or during the working day. The programme was led and managed by a third sector organisation based in the UK (referred to as the lead organisation), commenced in January 2008 and was completed in May 2012. Five workplaces from different sectors and locations in England were recruited to take part in the employers’ scheme which was part of the Walking Works programme. As part of the employers’ scheme, volunteer employees were recruited from within each workplace to act as ‘walking champions’. The walking champions were typically those who had a role in sustainable travel or health promotion within their workplace. They took an active role in planning and delivering activities, with support from the lead organisation, in order to gain experience to enable them to continue promoting walking in their workplaces beyond the funded programme.

The intended implementation strategy included a number of key features: 1) engagement of senior management to support the implementation of the programme and embedment into the workplace; 2) creation of a programme steering group; 3) recruitment of a network of walking champions (one champion for every 25 employees); 4) the expectation that each walking champion would spend five hours per month on the programme; 5) the development of a programme delivery plan by each workplace in consultation with the lead organisation; 6) delivery of eight activities in each workplace over the two years of the programme; and 7) provision of £1000 per year for each workplace to support programme activities. There was no standardised programme of activities for Walking Works; however, a menu of options was provided with suggestions for activities which could be delivered (see Additional file 1). Walking champions selected activities based on the interests of their workplaces and developed some of their own activities. Taking part was free and all employees were eligible to participate in the activities which were delivered in their workplace; there were no inclusion or exclusion criteria and there was no overall sign-up or registration process for the programme. Concurrent to the employers’ scheme, a national Walking Works campaign was delivered through a website (no longer available) that: provided tools and resources aimed at employees and employers demonstrating the health, well-being and other benefits of regular walking; allowed employees to ‘pledge’ to walk more; and provided employers with resources to develop their own walk to work schemes. An annual ‘Walk to Work Week’ was also held in May of each year to challenge employers and employees to increase the amount of walking they do on their daily commute, supported by an additional on-line tool, resources, challenges and competitions. Workplaces taking part in the employers’ scheme were able to use the resources in the national campaign and take part in Walk to Work Week.

### Data collection

Data were collected at the individual (employee) level and the organisational (workplace) level. Individual level data were collected using two cross-sectional online surveys (baseline and follow-up) which were conducted with employees in all participating workplaces. Baseline data were collected as soon as possible after the workplace had been recruited and before activities commenced (December 2009 to June 2010). Follow-up data were collected at the official end of the programme (September to November 2011). The specific dates when survey data collection took place in each workplace are provided in Table 1. All employees in the five participating workplaces were invited to take part in each survey via e-mail and other methods usually used by each workplace for communicating with staff (e.g. using pay slips, or via a line manager at team meetings). The baseline survey was sent to 5512 employees, the follow-up survey was sent to 4329 employees. The majority of employees received an invitation to complete both surveys, with the exception of those who left or joined the organisation before follow-up. There were fewer employees overall at follow-up due to organisational changes which led to reduced numbers of staff.

**Table 1:** Overview of Walking Works workplaces, programme activities and participation

	Workplace				
Region	North East	East Midlands	Yorkshire	West Midlands	London
Type	Further education institution	Private organisation	NHS organisation	County Council	Higher education institution
Dates of baseline survey	Start: 07–12-09 End: 16–12-09	Start: 20–04-10 End: 10–05-10	Start: 19–01-10 End: 10–03-10	Start: 14–05-10 End: 25–06-10	Start: 02–02-10 End: 02–03-10
Total number of employees (baseline)	400	1734	1778	1100	500
Number of employees (%) completing baseline survey	98 (25%)	653 (38%)	285 (16%)	434 (39%)	74 (15%)
Dates of follow-up survey	Start: 12–09-11 End: 04–10-11	Start: 02–11-11 End: 28–11-11	Start: 16–09-11 End: 07–11-11	Start: 29–09-11 End: 20–10-11	Start: 05–09-11 End: 10–10-11
Total number of employees (follow-up)	400	1729	300	1100	500
Number of employees (%) completing follow-up survey	144 (36%)	587 (34%)	59 (20%)	103 (9%)	25 (5%)
Workplace recruitment to intervention	Lead organisation contacted the walking champion to discuss taking part through a local travel plan contact.	Lead organisation promoted the intervention through a travel planning network. The walking champion expressed interest in taking part.	Lead organisation contacted the walking champion to ask for suggestions for other workplaces to take part. Workplace keen to take part themselves.	Heard about Walking Works through lead organisation’s website and contacted them.	Walking champion was referred through lead organisation’s member of staff not involved in the intervention.
Reasons for participation	The programme supported work on health and staff retention agendas, in particular looking at how to get people to and from work following move to multiple sites with restricted parking.	The programme supported work on health and travel agendas. Workplace looking for ways to help people become less dependent on cars and support mileage and carbon reduction targets.	The programme supported work on health and travel agendas. Particularly looking at ways to reduce carbon footprint and promote active travel across the workplace and their partners.	The programme linked to walking champion core role in sustainable travel.	The programme supported walking champion in core role to improve environmental performance of workplace. Also compatible with health and travel agendas: reducing environmental impact, carbon footprint and increasing staff awareness of these issues.
Links to existing workplace policy, strategy and programmes	Linked to workplace’s health at work policy and the strategic direction of the workplace to employ and retain the best staff. Strategy in place to ensure co-ordinated approach to travel planning; each site has a travel plan including a communication strategy to inform employees of alternatives to using car. Fitted with workplace’s existing health and well-being programme.	Programme linked to sustainable travel and well-being agendas across the workplace.	Linked to workplace health policy and sustainable travel plans and aspiration of workplace to be a local leader in health and healthy transport.	Linked to the sustainable travel agenda.	Programme linked to staff travel plans and health and well-being agendas.
Activities delivered	• Walk to Work Week 2010 and 2011 • Mince Pie Calculator promotion^a^ (Christmas 2010) • Led lunchtime walks • Alternatives to the car – staff conference (June 2010) • Fit Campaign – existing initiative (including walking)	• Walk to Work Week 2011 • Mince Pie Calculator promotion^a^ (Christmas 2010) • Unleash Your Office Animal quiz promotion^b^ (Summer 2010) • Photo competition^c^ (Spring 2011)	• Mince Pie Calculator promotion^a^ (Christmas 2009 and 2010) • Lunchtime led walks • New Year, New You staff travel days promotions (Jan-Feb 2010 and 2011) • Best Foot Forward pedometer challenge (April–May 2010 and May–June 2011)	• Walk to Work Week 2011 • Lunchtime led walks • Autumn pedometer challenge (October 2010) • Winter Warmers (Winter 2010) • Walking talk and quiz (February 2011)	• Walk to Work Week 2010 and 2011 • Lunchtime walks 2010 • Walking Works campaign launch lunch and walk • Walking champions meeting (Spring 2010) • Summer Social Walk 2010 • Foot pamper day (Sept 2011)
n (%) unaware of programme	20 (13.9%)	379 (64.6%)	11 (18.6%)	20 (19.4%)	8 (32.0%)
n (%) aware of programme (no participation)	62 (43.1%)	119 (20.3%)	12 (20.3%)	61 (59.2%)	5 (20.0%)
n (%) participated in programme(aware and participated in at least 1 activity)	62 (43.1%)	89 (15.2%)	36 (61.0%)	22 (21.4%)	12 (48.0%)
Frequency of participation n (%)	0 activities = 82 (56.9%) 1 activity = 28 (19.4%) 2 activities = 24 (16.7%) 3 activities = 3 (2.1%) 4 activities = 2 (1.4%) >5 activities = 5 (3.5%)	0 activities = 498 (84.8%) 1 activity = 76 (12.9%) 2 activities = 8 (1.4%) 3 activities = 5 (0.9%) 4 activities = 0 (0.0%) >5 activities = 0 (0.0%)	0 activities = 23 (39.0%) 1 activity = 17 (28.8%) 2 activities = 10 (16.9%) 3 activities = 4 (6.8%) 4 activities = 2 (3.4%) >5 activities = 3 (5.1%)	0 activities = 81 (78.6%) 1 activity = 17 (16.5%) 2 activities = 3 (2.9%) 3 activities = 1 (1.0%) 4 activities = 1 (1.0%) >5 activities = 0 (0.0%)	0 activities = 13 (52.0%) 1 activity = 3 (12.0%) 2 activities = 3 (12.0%) 3 activities = 1 (4.0%) 4 activities = 3 (12.0%) >5 activities = 2 (8.0%)

^a^ Mince Pie Calculator promotion was a free online tool which enabled employees to turn minutes walked into calories burnt (or equivalent to the number of mince pies eaten) to promote walking during the Christmas period

^b^ Unleash Your Office Animal quiz was an online quiz which asked questions about physical activity in relation to the working day. Depending on the answers given, the individual was assigned an office animal that represented their level of activity and provided suggestions on how they could increase their activity through walking.

^c^ Photo competition: employees were asked to take a photograph on their walk to work responding to the question “what is the thing you look forward to on your way to work” and an online gallery was created. Prizes were offered in the form of vouchers.

The surveys assessed usual mode of travel to and from work, time spent walking on the journey to and from work, time spent walking during the working day and potential mediators of behaviour change identified from the Theory of Planned Behaviour [20] (e.g. perceived behavioural control, intention and social norms). Self-reported physical activity was assessed using a single item measure of physical activity which asked “In the past week, on how many days have you done a total of 30 minutes or more of physical activity which was enough to raise your breathing rate? This may include sport, exercise, and brisk walking or cycling for recreation or to get to and from places, but should not include housework or physical activity that may be part of your job.” [21]. Work-related physical activity was assessed using a question taken from the European Prospective Investigation in Cancer and Nutrition questionnaire (EPIC) [22] which stated “We would like to know the type and amount of physical activity involved in your work. Please tick the option that best corresponds with your occupation(s) from the following four possibilities: sedentary occupation (you spend most of your time sitting, such as in an office); standing occupation (you spend most of your time standing or walking, however, your work does require intense physical effort (e.g. shop assistant, hairdresser, guard)); manual work (this involves some physical effort including handling of heavy objectives and use of tools (e.g. plumber, electrician, carpenter, cleaner)); heavy manual work (this implies very vigorous physical activity including handling of very heavy objects (e.g. dock worker, miner, bricklayer, construction worker)). Respondents also reported their individual characteristics including: gender, age, ethnic group and highest educational qualification. In addition, work-related characteristics were reported including: distance lived from work, occupational classification (selected from: senior managers or directors, middle or junior managers, traditional professional occupations, modern professional occupations, clerical and administrative occupations, technical and craft occupations, or semi-routine manual and service occupations) and working hours (full-time or part-time employment). Awareness of, participation in and perceptions of activities were assessed in the follow-up survey only.

Organisational (i.e. workplace) level data were collected at follow-up through telephone interviews with key personnel involved in implementing the programme. The lead organisation co-ordinator, all walking champions (*n* = 8) and a business representative from each organisation (*n* = 5) were invited to take part in a telephone interview. A semi-structured interview guide was used to initiate and direct the discussions through theme areas including: roles and responsibilities; programme management; organisational engagement and support; development and implementation; challenges and successes; impact; and sustainability. Interviews lasted 30–45 min and were recorded with the interviewee’s agreement.

### RE-AIM evaluation

A summary of the RE-AIM indicators assessed in this evaluation and the data sources used is provided in Table 2.

**Table 2:** Assessment of RE-AIM indicators

Indicator	Data source
Reach	
* An individual measure of participation and participant characteristics along with an assessment of representativeness of participants compared to non-participants.*	
• Awareness and participation in walking activities	Follow-up survey
• Differences between respondents based on awareness and participation in intervention activities for:	Follow-up survey
- individual characteristics (e.g. gender, age, ethnic group)	
- work-related characteristics (e.g. distance lived from work, occupation, physical activity at work)	
- meeting physical activity recommendations	
- usual mode of travel to work	
- time spent walking on the journey to/from work	
- time spent walking during the working day	
Effectiveness	
* Individual measures of the positive and negative consequences of the programme including behavioural, quality of life and participant satisfaction outcomes.*	
• Usual mode of travel to work	Baseline and follow-up survey
• Time spent walking for some or all of the journey to/from work	Baseline and follow-up survey
• Time spent walking during the working day	Baseline and follow-up survey
• Perceived change in frequency of walking to and from work in the last 18 months	Follow-up survey
• Perceived change in frequency of walking during the lunch break in the last 18 months	Follow-up survey
• Perceived change in frequency of walking at work in the last 18 months	Follow-up survey
• Perceived benefits to physical activity levels and health	Follow-up survey
Adoption	
* Organisational measures of the proportion and representativeness of settings that adopt the programme and barriers to adoption.*	
• Number of workplaces recruited	Lead organisation co-ordinator interview
• Characteristics of workplaces recruited	Walking champion interviews
Implementation	
* Organisational measures of the extent to which the programme is delivered as intended; individual measures of participant adherence.*	
• Organisational and senior management support	Walking champion interviews Business representative interviews
• Delivery of the intervention as intended including use of walking champions and planning and delivery of walking activities.	Lead organisation co-ordinator interview Walking champion interviews Business representative interviews
• Participant adherence (number of activities participants took part in)	Follow-up survey
• Perceptions of intervention activities	Follow-up survey
• Perceived encouragement for walking on the journey to and from work	Follow-up survey
• Perceived encouragement for walking during the working day	Follow-up survey
• Likes and dislikes of intervention activities and suggestions for improvement	Follow-up survey
Maintenance	
* Assessment of long term maintenance of change at the individual level (sustained change in behaviour) and at the organisational level (the extent to which the programme becomes routine/embedded in the everyday culture and norms of an organisation).*	
• Plans for the sustainability of the intervention activities	Walking champion interviews Business representative interviews
• Confidence to include some walking as part of the journey to or from work on most days	Follow-up survey
• Intention to walk to work on a regular basis in the next few months	Follow-up survey
• Encouragement needed to walk all or some of the journey to and from work	Follow-up survey
• Encouragement needed to walk during the working day	Follow-up survey

### Reach

Assessment of programme reach was based on those who completed the follow-up survey and reported awareness or participation in programme activities. The follow-up survey was tailored for each workplace and included a pre-defined list of walking activities which had been delivered as part of the programme in the relevant workplace. The list of activities was provided to the research team by the walking champions and confirmed by the lead organisation co-ordinator. Employees were asked to indicate which activities they were aware of or had participated in. From this they were classified into one of two groups: ‘unaware’ of the programme or ‘aware’ of the programme (aware of or participated in at least one activity). Representativeness was assessed by comparing the individual characteristics, employment-related characteristics and physical activity levels of those who were unaware of the programme activities with those who were aware of the programme. A similar approach for assessing representativeness has been used elsewhere [23, 24].

### Effectiveness

Changes in the outcome measures were assessed by comparing responses in the baseline and follow-up surveys. In addition, differences in the outcome measures between those who participated in programme activities and those who did not participate were compared. The primary outcome measures were the proportion of employees walking for all, or some, of their journey to/from work, time spent walking on the journey to/from work, time spent walking during the working day and the proportion of employees undertaking incidental walking at work. Secondary outcomes focused on mediators of behaviour change and included confidence (perceived behavioural control), intention and colleague support (social norms) for walking to/from work and walking during the working day.

#### Walking to and from work

Respondents were asked to complete a one week travel diary indicating which modes of transport they had used to travel to and from work for each day in the last week. The travel diary has been shown to have acceptable test-retest reliability [25]. Respondents were given the option of seven modes including: walking; bicycle; car, taxi or van; bus or coach; rail, tram or underground; motorcycle or moped; and other, and were asked to indicate all modes of transport used. In addition, respondents reported the number of minutes they spent walking to and from work separately for each day in the last week (week and weekend days). As Government recommendations for physical activity suggest that bouts of 10 min of activity are needed to benefit health [26], any journeys lasting less than 10 min were recoded as 0 min and the corresponding walking trips were removed from the travel diary. Responses to the journey to work and the journey from work in the travel diary were recoded separately for each day into the following five categories: walking only; walking and other mode(s) (including car or public transport); cycling; motorised transport; and public transport. The most frequently reported mode across all days was recorded as being the respondent’s usual mode of transport. Where respondents reported equal numbers of days using the same mode (*n* = 22), the least active mode was selected as the usual mode. The total number of minutes spent walking to and from work in the last week was computed by summing the number of minutes walking reported for each day in the last week. Respondents were then categorised as to whether they walked for 0 min per week, 1–100 min per week or >100 min per week on their journey to and from work.

#### Walking during the working day

The time spent walking during the working day was assessed using a question from the long version of International Physical Activity Questionnaire (IPAQ) [27]: “During a usual week, on how many days do you walk for at least 10 minutes as part of your work? Please do not include any walking you do as part of your journey to and from work.” Respondents who answered one or more days were also asked “How much time do you usually spend walking as part of your work on one of those days?”. Responses were categorised into walking 0 min per day, 1–30 min per day or >30 min per day. Respondents were also asked about incidental walking regarding how often they participate in the following activities at work: a) climb the stairs instead of using the lift; b) walk to talk to a colleague instead of using e-mail or the telephone; c) walk for at least 10 min to get to or from a business meeting; d) take part in a walking meeting; and e) walk for at least 10 min at lunchtime. Response options were on a four point Likert scale of “never/rarely”, “some days”, “most days” or “every day”.

#### Mediators of behaviour change

Respondents were asked to what extent they agreed that a) I am confident that I can include some walking as part of my journey to or from work on most days (perceived behavioural control); and b) I intend to walk for all or part of my journey to or from work on a regular basis in the next few months (intention). Responses were on a four point Likert scale from “strongly disagree” to “strongly agree”. Respondents were also asked about colleague support for walking using the question “During the past month, how much have your work colleagues encouraged you to a) walk for some or all of your journey to or from work; b) hold a walking meeting; and c) go for a walk at lunchtime (social support). Response options were on a five point Likert scale from “never” to “very often”.

#### Perceived impact of programme activities

Perceived impact of the programme activities on walking levels, physical activity and health was assessed in the follow-up survey only. Respondents were asked to consider the last 18 months and report to what extent they agreed with the statements “I have walked for all or part of my journey to and from work more often”; “I have walked during my lunch break more often” and “I have walked at other times during my working day more often”. Those who participated in the activities were compared to those who did not. Respondents who participated in the activities were also asked about the perceived benefits of the activities for their health (physical activity levels, general health, weight loss and stress levels). Response options were on a four-point Likert scale from “strongly disagree” to “strongly agree”.

### Adoption

The lead organisation’s co-ordinator provided the research team with details about how workplaces were recruited for the programme, how many workplaces had been recruited and the challenges and successes of recruiting workplaces. Details about organisational characteristics, such as number of employees, how organisations heard about the programme, reasons for participation and links to existing workplace policy, strategy and programmes were obtained from interviews with walking champions and business representatives.

### Implementation

Interviews with key personnel were used to assess the implementation of the programme and which aspects were delivered as intended. Participation and adherence (defined as participants who took part in multiple activities) were measured using the number of activities respondents reported taking part in, which was assessed in the follow-up survey. In addition, survey respondents who were aware or participated in programme activities were asked to indicate to what extent they agreed the activities had been well publicised and were convenient to join. For those who participated, respondents were asked to what extent they agreed the activities were enjoyable, were informative, met their needs, had encouraged them to walk more on their journey to and from work and encouraged them to walk more during their working day. Response options were on a four-point Likert scale from “strongly disagree” to “strongly agree”. Finally, respondents were provided with an open response question asking them to comment on what they liked and disliked about the activities and to make suggestions for improvements.

### Maintenance

Interviewees were asked about whether the programme had been integrated into their workplaces and the sustainability of programme activities, including any further funding being provided. Data reported in the follow-up survey were used to compare confidence and intention to walk for all, or part, of the journey on a regular basis in future, between those who participated in activities and those who did not. Finally, survey respondents were asked two open ended questions: “What would encourage you to walk for all or some of your journey to and from work?” and “What would encourage you to walk more during your working day, either at break times or as part of your work?”.

### Data analyses

Descriptive data were summarised using percentages. Data collected from baseline and follow-up employee surveys were used to assess effectiveness and were treated as independent samples. In addition, differences in primary and secondary outcomes were assessed by comparing those who participated in programme activities with those who did not participate. For categorical data, Chi square tests were conducted assessing differences between baseline and follow-up surveys and between participants and non-participants. Continuous data were analysed to test for significant differences over time using an independent t-test. Where data were not normally distributed, non-parametric tests (Mann-Whitney U test) were utilised. The follow-up survey only was used to assess the other domains of the RE-AIM framework. Chi square tests were conducted to assess differences between groups. Data were analysed in SPSS Statistics (version 22.0) (IBM SPSS Inc., Armonk, New York). Responses to open ended survey questions were reviewed and the most frequently mentioned comments identified.

All interviews were transcribed verbatim by an independent administrator. Transcripts were read to understand participants’ perspectives, initial coding was undertaken in NVivo 10 to group findings into themes related to the interview guide and further coding was undertaken to identify the themes related to the overall implementation of the Walking Works programme. Key points were extracted and information presented in relation to the three organisational level dimensions of the RE-AIM framework: adoption, implementation and maintenance.

## Results

Overall, 1544 employees completed the baseline survey (28% response rate) and 918 employees completed the follow-up survey (21% response rate). Twelve telephone interviews were conducted with the lead organisation’s co-ordinator, eight walking champions and three senior business representatives. Results for the RE-AIM domains are presented in the order adoption, reach, implementation, effectiveness and maintenance to reflect the logical process in which programme delivery takes place [28].

### Adoption

The lead organisation’s co-ordinator indicated that a variety of approaches were used to engage with workplaces e.g. via the lead organisation’s website, e-bulletin, cold calling and using existing networks as well as other regional and national external networks. Recruitment was reported to be more challenging and took longer than envisaged as whilst there was interest from workplaces, many were unable to commit resources for the duration of the programme and some were not able to fulfil monitoring and evaluation requirements. Five workplaces from different sectors and settings across five regions of England agreed to take part (Table 1). At baseline the number of staff employed in each organisation ranged from 400 to 1778. Workplaces were situated in a variety of locations with varying pedestrian access and road networks, and mixed availability of public transport and car parking. All workplaces had an existing sustainable travel plan and walking champions and business representatives indicated the reasons for taking part were that workplaces were keen to encourage their staff to be less dependent on cars, wanted to reduce their carbon footprint, were interested in promoting health and well-being in their employees or the programme was thought to fit with the existing role of the champion. All workplaces remained engaged until the official end of the programme, despite organisational changes and a challenging economic climate at the time of delivery. Details of the workplaces which declined to take part were not collected therefore it is difficult to make any assessment of the representativeness of the workplaces which participated.

### Reach

Of the 918 employees who responded to the follow-up survey, 47.7% (*n* = 438) were unaware of the activities and 52.3% (*n* = 480) were aware of or participated in at least one of the activities delivered. There were significant differences in characteristics between the two groups in gender, age, educational qualifications, occupation and work-related physical activity (Table 3). A higher proportion of those aware of the activities were female, aged 30 or older, had a University degree and had a professional occupation compared to those who were unaware of the activities; and a lower proportion of those aware of activities had a sitting occupation. In addition, a significantly higher proportion of those aware of the activities walked during the working day.

**Table 3:** Characteristics of survey respondents by awareness of programme activities

Characteristic	Unaware of programme activities		Aware of programme activities		
	*n* = 438		*n* = 480		
	n^a^	%	n^a^	%	*p*
Gender					
Female	202	60.3	324	69.4	**0.008**
Age (years)					
16–30	121	36.7	126	27.5	**<0.001**
31–44	144	43.6	187	40.8	
≥ 45	65	19.7	145	31.7	
Ethnicity					
White	300	90.1	430	93.3	0.132
Highest educational qualification					
University degree	97	30.9	226	51.4	**<0.001**
Higher education/certificate	34	10.8	55	12.5	
GCE ‘A’ Level	97	30.9	95	21.6	
GCSE Grades A to C	86	27.4	64	14.5	
Distance live from work					
≤ 2 miles	62	15.2	93	19.5	0.263
2.1–5 miles	120	29.4	129	27.0	
5.1–10 miles	125	30.6	129	27.0	
> 10 miles	101	24.8	126	26.4	
Occupation					
Senior or Middle Manager	61	17.9	89	18.9	**<0.001**
Professional occupation	33	9.7	134	28.5	
Clerical	237	69.5	236	50.2	
Working hours					
Full-time	264	77.4	373	79.9	0.399
Part-time	77	22.6	94	20.1	
Work-related physical activity					
Sitting occupation	399	95.2	414	87.0	**<0.001**
Physical activity levels					
Meeting current recommendations^b^	87	26.0	94	20.1	0.051
Usual mode of travel to work					
Walking only (≥10 min)	41	10.6	44	9.5	0.114
Walking (≥10 min) and other mode	82	21.2	101	21.7	
Cycling	8	2.1	20	4.3	
Public transport	27	7.0	18	3.9	
Motorised transport	229	59.2	282	60.6	
Walking to/from work					
0 min per week	156	49.8	187	49.7	0.473
1–100 min per week	62	19.8	87	23.1	
> 100 min per week	95	30.4	102	27.1	
Walking at work					
0 min per day	95	36.1	81	25.6	**0.013**
1–30 min per day	112	42.6	144	45.6	
> 30 min per day	56	21.3	91	28.8	

^a^ Numbers do not sum up to total due to missing responses

^b^ Assessed using a single item measure of physical activity [21]

Bold numerical values: *p*=<0.05

### Implementation

Three themes emerged from the interviews with walking champions and business representatives relating to implementation. These included: organisational and senior management support; use of walking champions and planning and delivery of walking activities.

#### Organisational and senior management support

Walking champions were encouraged to engage senior management in the programme to help link the programme with broader business objectives, to lever internal resources and support and to try and embed activities into normal daily practice. Champions from three workplaces reported that senior level staff supported the programme, although visible participation of senior staff in activities was only reported in two workplaces suggesting buy-in to the programme may have been low. It was also recommended that a steering group was set up within the workplaces to support the programme. None of the workplaces did this but some linked into existing, related steering groups, e.g., travel planning or health and well-being and aligned the programme with broader existing activities.

#### Use of walking champions

It was initially planned that a network of employee walking champions would be recruited within each workplace with one champion for every 25 employees. However, the lead organisation co-ordinator reported that, in practice, each participating workplace only had one or two champions who led and acted as the main contact for the programme. In total, eight volunteer walking champions were recruited across the five workplaces; two workplaces had one champion and three workplaces had two champions. The champion for one workplace reported that they had attempted to set up a network of walking champions. However, it proved challenging to involve them in programme delivery due to competing demands from their normal daily roles.

The normal role of walking champions varied although seven of them had roles relating to sustainable/active travel. Walking champions found it easier to engage with the programme where the role was closely aligned to their normal daily job requirements. The main role of the walking champions in the Walking Works programme was to plan and deliver activities to promote walking with support from the lead organisation. The intention was for walking champions to progress to taking a lead in developing ideas and implementing activities themselves as they gained more experience to ensure the sustainability of activities beyond the end of the programme. Champions had a variety of skills and experience which resulted in varying levels of support being requested from the lead organisation to deliver activities. The lead organisation co-ordinator stated that some champions requested support with research and resources, others requested more hands on support to help them organise events and undertake promotional work. Key attributes identified by walking champions for their role were motivation, enthusiasm, assertiveness, positivity, creativity, being organised, flexibility and persistence.

Walking champions were asked to spend 5 h per month on the Walking Works programme. The actual time spent varied across workplaces ranging from 1 h per week to 1 day per week. As the programme was part of the walking champion’s broader work it was not always possible to prioritise programme activities. Lack of senior management involvement and insufficient support on delivery of activities were mentioned as challenges for the walking champions in undertaking their role.

#### Planning and delivery of walking activities

The workplaces commenced implementing activities between December 2009 and June 2010 and continued for 18 to 22 months when funding for the overall programme ceased. The lead organisation co-ordinator reported that there were a number of challenges in delivering what was originally planned for the programme with what was possible to deliver in the workplaces. Walking champions had many competing priorities and the lead organisation co-ordinator reported that a flexible, pragmatic approach had to be taken to maintain the engagement of the recruited champions. The lead organisation held an initial meeting with each participating workplace to discuss the walking activities that might be delivered, after which workplaces were expected to put together a formal plan of activities for the duration of the programme with clear milestones and timescales. Only two of the workplaces developed such a plan, the others relied on the lead organisation to develop a plan for them.

The initial plan was for each workplace to deliver eight activities over two years. This target was later reduced to take into account walking champion’s ability and capacity to deliver activities alongside their other work commitments. Four of the five workplaces delivered all the activities which were discussed at the initial meeting with the lead organisation. A variety of activities were delivered across the workplaces (Table 1) with most taking part in national campaigns (e.g. ‘Walk to Work Week’). Other activities included lunchtime walks, a staff conference with a specific focus on using alternatives to the car and team pedometer challenges. Champions also created their own activities which were not listed on the menu of options. Walk to Work Week was mentioned most frequently as a success of the programme. Overall, walking champions were positive in terms of how they felt the programme had been implemented in their workplaces and the value of the support and resources the lead organisation provided. Many of the workplaces underwent or initiated restructuring and/or relocation during the programme period which may have impacted on the delivery of activities and employee engagement.

Each workplace was offered up to £1000 in both years of the programme to deliver activities; this funding was claimed by four of the five workplaces. The other workplace had an internal budget available (amount not known) so did not make any claims. The funding was thought to be sufficient to at least start the programme. However, some walking champions thought additional funding would have been useful to develop some of their ideas further and produce resources which might have a longer lasting impact, e.g., walking maps. The ‘ready to use’ resources provided in the Walking Works programme, such as for Walk to Work week, were welcomed by the walking champions as they were easy to implement and therefore facilitated delivery of activities in their workplaces. In contrast, insufficient funding or resources and having to adhere to national timescales for delivering activities, which sometimes coincided with other work commitments, were mentioned as challenges to delivery.

A variety of forms of communication were used to promote walking activities including posters, notice boards, digital display screens, weekly staff magazine, staff newsletter, road shows, intranet, all staff e-mails and 1:1 contact either face to face, by telephone or e-mail. Four of the eight walking champions identified individual e-mail as the most successful method for reaching and engaging participants. The champions reported that organisational support for communicating information about the activities varied.

#### Participation, adherence and participant perspectives

Overall, 24.1% (*n* = 221) of survey respondents reported participating in at least one of the activities provided. Adherence to the programme varied with 15.4% (*n* = 141) of respondents taking part in one activity, 5.2% (*n* = 48) taking part in two activities, 1.5% (*n* = 14) taking part in three activities, 0.9% (*n* = 8) taking part in four activities and 1.0% (*n* = 10) taking part in five or more activities. Within each workplace, individual levels of participation varied (Table 1).

There were mixed views about the activities which were delivered. Of those who were aware or participated, 58.9% agreed activities were well publicised and 49.5% agreed activities were convenient to join. In those who participated, 58.9% agreed activities were enjoyable; 64.8% agreed activities were informative; 52.1% agreed activities met their needs; 44.7% agreed the activities had encouraged them to walk more on their journey to and from work and 64.8% agreed the activities had encouraged them to walk more during their walking day. The most frequently mentioned dislikes about the programme were lack of publicity for activities; work commitments and a lack of time which prevented respondents taking part in the activities. Suggested improvements for the programme included improved publicity and more visible support from senior management in the workplaces for walking activities.

### Effectiveness

Individual and workplace-related characteristics of survey respondents at baseline and follow-up are presented in Additional file 2. There were no significant differences in respondent characteristics between the baseline and follow-up surveys with the exception of distance lived from work, with fewer respondents to the follow-up survey living ≤2 miles away. The proportion of respondents travelling by different modes of transport at each survey time point is shown in Table 4. Use of motorised vehicles such as cars was high in both surveys (baseline: 61.0%; follow-up: 60.0%). The proportion of participants who only travelled by walking for their journeys was higher at baseline (baseline: 11.2%; follow-up: 10.0%) but a higher proportion of participants walked for some of their journey in combination with using other modes at follow-up (baseline: 20.6%; follow-up: 21.5%). There were no significant differences (*p* = 0.461) in usual mode of travel to and from work between baseline and follow-up. There were also no significant differences between baseline and follow-up in time spent walking on the journey to and from work, walking during the working day, incidental walking (with the exception of walking at lunchtime, which was significantly lower at follow-up), confidence and intention to walk for all or part of the journey to work, or colleague support for walking to/from work, holding walking meetings or going for a walk at lunchtime (Table 4).

**Table 4:** Changes in walking levels between baseline and follow-up surveys

		Baseline	Follow-up	
		% (n)	% (n)	p
Usual mode of transport to and from work	Motorised transport	61.0 (860)	60.0 (511)	0.461
	Public transport	3.8 (54)	5.3 (45)	
	Cycling	3.3 (47)	3.3 (28)	
	Walking (≥10 min and other mode)	20.6 (290)	21.5 (183)	
	Walking only (≥10 min)	11.2 (158)	10.0 (85)	
		% (n)	% (n)	p
Time spent walking to and from work	0 min per week	50.6 (602)	49.8 (343)	0.799
	1–100 min per week	22.2 (264)	21.6 (149)	
	>100 min per week	27.2 (323)	28.6 (197)	
Time spent walking during the working day	0 min per day	26.8 (276)	30.4 (176)	0.100
	1–30 min per day	43.1 (443)	44.2 (256)	
	>30 min per day	30.1 (309)	25.4 (147)	
Incidental walking		% most/every day (n)	% most/every day (n)	p
Climb stairs instead of using lift		74.1 (1044)	74.9 (655)	0.652
Walk to talk to colleague		58.9 (860)	59.6 (529)	0.749
Walk for at least 10 min to get to a business meeting		12.8 (170)	13.3 (108)	0.747
Take part in a walking meeting		1.2 (15)	1.3 (10)	0.883
Walk for at least 10 min at lunchtime		39.2 (579)	30.7 (272)	**<0.001**
Mediators of behaviour change		% agree (n)	% agree (n)	p
Confidence to include some walking as part of my journey to or from work on most days		50.5 (694)	53.7 (453)	0.135
Intention to walk for all or part of my journey to or from work on a regular basis in the next few months		41.5 (564)	42.3 (354)	0.714
Colleague support in the last month		% often/very often (n)	% often/very often (n)	p
Walking for some or all of the journey to or from work		4.3 (61)	5.8 (50)	0.110
Holding a walking meeting		0.9 (13)	1.5 (13)	0.201
Going for a walk at lunchtime		14.8 (208)	15.6 (133)	0.638

Bold numerical values: p=<0.05

Differences between participants and non-participants were also compared for the primary and secondary outcomes (Table 5). There were no significant differences between groups for usual mode of transport to and from work, time spent walking to and from work, or time spent walking during the working day. Participants reported significantly higher levels of walking at lunchtime most or every day compared to non-participants. Compared to those who did not participate in the activities, a significantly higher proportion of respondents who participated agreed that, in the last 18 months, they had walked more often for all or part of their journey to and from work, during their lunch break and at other times during their working day (Table 5). Some participants agreed the activities had helped them to be more physically active (53.0%), made them feel healthier (55.0%), helped them lose weight (35.2%) and helped them feel less stressed (52.1%).

**Table 5:** Walking levels in non-participants and participants^a^

		Non-participants	Participants	
		% (n)	% (n)	p
Usual mode of transport to and from work	Motorised transport	61.3 (391)	56.1 (120)	0.160
	Public transport	5.8 (37)	3.7 (8)	
	Cycling	2.8 (18)	4.7 (10)	
	Walking (≥10 min and other mode)	21.2 (135)	22.4 (48)	
	Walking only (≥10 min)	8.9 (57)	13.1 (28)	
		% (n)	% (n)	p
Time spent walking to and from work	0 min per week	51.5 (261)	45.1 (82)	0.152
	1–100 min per week	21.9 (111)	20.9 (38)	
	>100 min per week	26.6 (135)	34.1 (62)	
Time spent walking during the working day	0 min per day	32.5 (139)	24.5 (37)	0.060
	1–30 min per day	44.4 (190)	43.7 (66)	
	>30 min per day	23.1 (99)	31.8 (48)	
Incidental walking		% most/every day (n)	% most/every day (n)	p
Climb stairs instead of using lift		73.2 (487)	80.4 (168)	**0.037**
Walk to talk to colleague		59.6 (399)	59.6 (130)	0.983
Walk for at least 10 min to get to a business meeting		11.9 (71)	17.5 (37)	**0.037**
Take part in a walking meeting		1.2 (7)	1.5 (3)	0.710
Walk for at least 10 min at lunchtime		30.1 (201)	32.3 (71)	0.551
Mediators of behaviour change		% agree (n)	% agree (n)	p
Confidence to include some walking as part of my journey to or from work on most days		52.0 (326)	58.8 (127)	0.084
Intention to walk for all or part of my journey to or from work on a regular basis in the next few months		39.7 (247)	49.8 (107)	**0.010**
Colleague support in the last 18 months		% often/very often (n)	% often/very often (n)	p
Walking for some or all of the journey to or from work		4.5 (29)	9.6 (21)	**0.006**
Holding a walking meeting		0.9 (6)	3.2 (7)	**0.019**
Going for a walk at lunchtime		14.8 (94)	17.9 (39)	0.271
Perceived changes in the last 18 months		% agreed (n)	% agreed (n)	p
Walked for all or part of the journey to and from work more often		34.6 (217)	46.3 (101)	**0.002**
Walked during the lunch break more often		50.8 (316)	64.8 (140)	**<0.001**
Walked at other times during the working day more often		49.2 (300)	61.0 (130)	**0.003**

^a^ Data from follow-up survey only

Bold numerical values: p=<0.05

### Maintenance

All workplaces remained engaged until the official end of the programme, despite reports from walking champions and business representatives regarding organisational changes and a challenging economic climate at the time of delivery. The business representatives and walking champions perceived that the programme had positively changed attitudes and behaviour towards walking to work and walking during the working day in their workplaces. Negative feedback included that many employees saw it as a one-off programme or challenge, rather than a long-term programme of activities to support behaviour change.

Walking champions and business representatives reported mixed plans for continued delivery of activities. All champions were keen for the activities to continue but only one workplace had secured funding for future activities as part of their travel planning and health and well-being programme. In another workplace there was a possibility of linking to occupational health activities and the champion was keen to roll out the activities to other sites in the workplace. One workplace planned to share their learning with other local workplaces, and two of their activities (pedometer challenge and road shows) were to be written into workplace’s annual business case due to their success. The remaining workplaces indicated activities would continue if funding could be identified. Interviewees suggested that in future the programme should aim to engage with core departments, e.g., human resources, occupational health and communications to help support delivery, integrate activities into normal daily business and promote sustainability.

Of those who participated in the programme activities, 58.8% were confident they could include some walking as part of their journey to or from work on most days (compared to 52.0% of those who did not participate; *p* = 0.084) and 49.8% intended to walk for all or part of their journey to or from work on a regular basis in the next few months (compared to 39.7% of those who did not participate; *p* = 0.010) (Table 5). In response to what participants thought would encourage them to walk for all, or some, of the journey to and from work, “nothing” was frequently stated along with a barrier to walking, such as living too far away from work, not having time or needing to drop children off at school. Other suggestions included: 1) providing incentives to walk, e.g., monetary, doing a charity event, competitions, dedicated walking weeks; 2) changing car parking arrangements, e.g., restricting access to car parks closer to work, and providing car parks further away so walking is required to get to the office; 3) providing pool cars for use at work; 4) changing the requirements for work, e.g., flexible working hours, time built into working day to allow for walking, less equipment to carry, not requiring a car for work, stable location for work, less work pressures, and a more relaxed dress code at work; 5) improving the environment, e.g., improved street lighting, better access to safe, familiar, well-lit areas, less pollution, quieter roads, a perception of safety, and improved gritting of pavements; 6) improvements in public transport e.g. staff bus service; and 7) having a walking buddy.

The most frequently mentioned comments relating to what would encourage participants to walk more during the working day, either at break times or as part of their work, included: 1) providing additional organised walking activities; 2) additional incentives to promote walking e.g. pedometers, or a points schemes with financial rewards; 3) having more time for breaks and longer breaks during the day; 4) changing the workplace culture and building walking into daily work activities (e.g. walking meetings); and 5) improvements to the physical activity environment in the workplace grounds and the local area around the workplace.

## Discussion

No differences were observed in walking to and from work and walking during the working day between baseline and follow-up, or between those who participated in programme activities compared to those who did not (assessed in the follow-up survey). This is in contrast to findings from reviews of workplace walking interventions [11–15]. Using the other domains of the RE-AIM framework helped to explain the absence of any changes which may be due to barriers faced in using employees (walking champions) to deliver the activities, implementation failure (a number of components of the programme were not delivered as they were originally intended), the types of activities which were delivered (many were short-term or one-off challenges or events) or poor publicity (there were low levels of employee awareness of the programme, and even lower levels of participation in activities, in those who completed the follow-up survey).

Walking champions faced challenges with their role in planning and delivering activities due to their capacity and competing demands of their normal daily job, their skills and lack of support from senior management. Therefore, if champions are to be used in future, the implementation of activities needs to be more fully integrated into their normal role. Similar findings with regards to alignment of roles have been reported for a workplace commuter cycling intervention [23]. In addition, having sufficient time, skills, knowledge and competence have been identified as important facilitators for the ‘implementer’ in workplace health promotion programmes [29]. The importance of strong senior management support in facilitating the delivery of workplace health promotion activities and active travel programmes has been reported elsewhere [29–31].

Awareness of the activities that had been delivered was low and many respondents thought the activities had not been well publicised. This suggests further work may be needed in relation to publicity and communication to reach all employees with information about the activities and opportunities to participate. Participation in activities was also low with only a quarter of survey respondents taking part, reflecting findings from elsewhere that less than 50% of employees typically take part in workplace health promotion programmes [32]. Additional insight is needed to develop activities which reach as many employees as possible and encourage participation, particularly for those who may be most in need [32]. In addition, many of those who were aware of or participated in the activities thought they had not been convenient to join and did not meet their needs. Assessing employee needs regarding the types and timing of activities, co-producing the programme with employee involvement and consultation with staff on an ongoing basis has previously been recommended for workplace physical activity programmes [16, 30].

Although a range of activities were delivered, they were mainly short-term and one-off individual or social approaches e.g. campaigns or walking groups. Many of the activities which were suggested or delivered were not evidence- or theory-based and had not been tested in a research environment. These may not have been sufficient to engage a high proportion of employees or instigate sustained behaviour change. In addition, a more comprehensive programme of individual, social, environmental, organisational and policy level changes may be needed to influence whole-workplace levels of walking. Although signposted on the menu of options, no environmental or policy changes, or attempts to change organisational culture (e.g. by introducing walking meetings), were reported during the programme. This may have been due to the level of influence of the walking champion or a lack of senior management support and engagement. It has also been reported elsewhere that changing the workplace environment and policy is difficult in the short-term and these types of changes should be considered as mid- to long-term objectives [30]. Employees did however suggest a number of strategies to support and encourage walking. The most frequently reported strategies to support or motivate walking to and from work included: providing incentives to walk; changing car parking arrangements; providing pool cars; changing workplace policy regarding work hours and dress code; improving the external physical environment in the areas immediately surrounding workplaces; and developing a walking buddy scheme. Strategies suggested to support or motivate walking during the working day included: providing additional walking activities and incentives; longer breaks; promoting walking meetings; and improving the external environment in the workplace grounds and local area. All these strategies warrant further investigation for use in whole-workplace walking programmes.

**Fig. 1:**
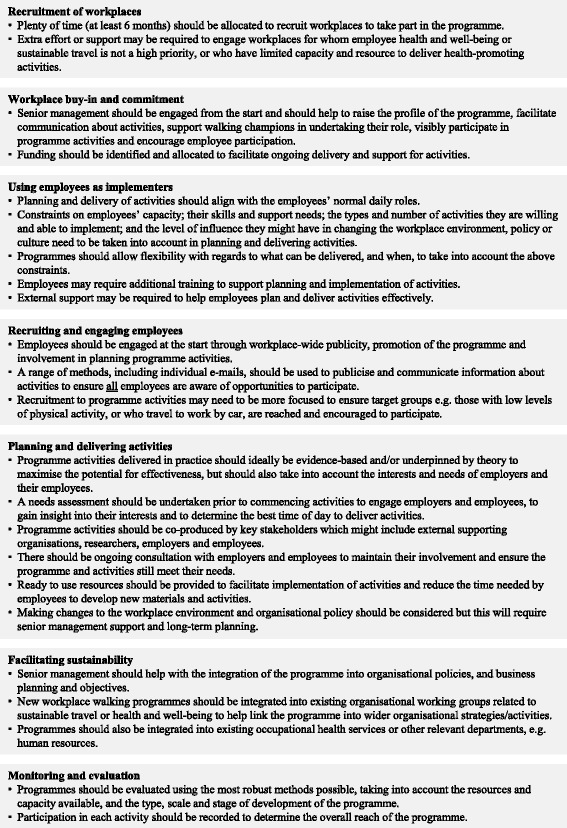
Recommendations for the implementation of whole-workplace walking programmes

Based on the findings from this study, a number of factors have been identified which should be taken into consideration and/or addressed by researchers and practitioners when planning and implementing employee-led, whole-workplace walking programmes. Recommendations for future programmes are outlined in Fig. 1.

### Strengths and limitations

A pragmatic evaluation of a whole-workplace walking programme which was planned and delivered by employees with support from an external organisation was undertaken. The strengths of the study included the use of an identical evaluation design in each workplace providing consistency in measures, and the use of employee surveys and interviews with those delivering the programme which helped to provide insight into implementation. Applying the RE-AIM framework [19] to examine the findings from the evaluation provided a useful approach for evaluating the Walking Works programme.

There were also some limitations for this study. Due to restrictions in the funding available for the evaluation, no control or comparison workplaces were included in the study design. In addition, data reported were from cross-sectional surveys and it was not possible to match participant’s data between baseline and follow-up to assess individual behaviour change. For both these reasons, the results relating to effectiveness should be interpreted with caution. The survey response was low meaning there is a high possibility of selection bias and, based on respondent characteristics, those who participated in activities may not have been representative of the employee population. Therefore, it may not be possible to generalise the findings. Self-report measures were used to assessing walking levels which may have resulted in over reporting of activity levels [33]. For some questions asked in the survey, only a single item from an instrument has been included. Although the validity for the whole instrument may have been demonstrated, the validity of individual items which have been extracted is not known and may not have been retained. Assessment of reach (awareness and participation in programme activities) was based on those who completed the follow-up survey and was low. It is possible that many more employees did take part in programme activities (but did not complete the follow-up survey) and interviews with walking champions and business representatives suggested this was the case. In contrast, participation in programme activities based on follow-up survey data may have been over-estimated, given that those who participated may have been more engaged and therefore more likely to complete the survey.

Evaluating real-world physical activity interventions is challenging. Whilst it is critical that more robust evaluation of practice-led interventions is undertaken to assess implementation and effectiveness, to improve programmes, and to facilitate scale-up, there are a number of barriers which need to be overcome in order to do this. Budgets provided for the evaluation of practice-led interventions are often small, with funders having unrealistic expectations of what can be achieved; timescales for developing evaluation methodology are often tight before intervention delivery commences, making it difficult to plan and integrate the evaluation effectively; interventions are often developed without input from researchers to ensure appropriate evidence, theories or frameworks are applied and/or tested; intervention delivery is often outside the control of researchers and can make using robust methodology difficult; and response rates to surveys are low as participants want to take part in the activity, but not be part of the research or evaluation. In order to address some of these issues it is important that researchers and practitioners work in partnership to co-produce interventions, and that the evaluation methodology is developed at the same time as the intervention is being planned. Funding appropriate to the type, scale and stage of development of the intervention should be sought to enable the most robust evaluation methodologies to be utilised, and time allowed during intervention planning for acquiring such funding. Evaluating both the implementation and the effectiveness of real world interventions will be essential in developing the evidence base for what works in promoting physical activity in real world settings.

## Conclusions

RE-AIM provided a useful framework for evaluating Walking Works, which was a practice-led, whole-workplace programme which aimed to promote walking for the journey to and from work and walking during the working day. No changes in walking behaviour were observed which may have been due to barriers in using employees to plan and deliver activities, some programme components not being delivered as intended, the types of activities delivered, or lack of awareness and participation by employees. If practice-led, whole-workplace programmes delivered by employees are to be successful there are a number of factors which need to be taken into consideration. Researchers and practitioners planning and implementing future whole-workplace walking programmes should consider the recommendations provided.

## Additional files

Additional file 1: Walking Works: menu of options for activities (PDF 98 kb)

Additional file 2: Employee characteristics at baseline and follow-up (PDF 170 kb)

## Abbreviations

RE-AIM: Reach, effectiveness, adoption, implementation, maintenance
