# Supplementary material for: Evaluation of the implementation of a whole-workplace walking programme using the RE-AIM framework
Source: BMC Public Health. 2017 May 18;17:466. doi: 10.1186/s12889-017-4376-7 (PMC5437663; doi:10.1186/s12889-017-4376-7)
Supplement: Supplementary file 1 — Walking Works: menu of options for activities (PDF 98 kb) [file 12889_2017_4376_MOESM1_ESM.pdf]

Additional File 1 Walking Works programme: menu of options for activities

Short-term promotions:

General publicity	• Hold a launch event to publicise Walking Works<br>• Send out regular e-communications to staff regarding walking and activity opportunities<br>• Interview staff walkers for intranets, websites, annual reports, newsletters, etc.
All walking	• Encourage employees to make a pledge on the Walking Works website in return for hints and tips to support walking via email<br>• Organise a 'walk doctor' session for staff (a consultation with a walking specialist to discuss walking habits, barriers to walking and identify solutions to encourage walking)<br>• Publicise charity walks and encourage staff to take part<br>• Link walking promotions to other promotions e.g. hold a Fairtrade breakfast for walkers during Fairtrade fortnight
Walking to work	• Take part in National Walk to Work Week<br>• Create an online chat with local transport experts (opportunity to raise concerns about routes)
Walking during work	• Organise lunchtime walks and talks (with an expert, person of interest?)<br>• Set up 'walk and talk with the Chief Executive' opportunities
Other	• Art attacks (e.g. turn a few car park spaces into chill out gardens)

Incentives and rewards:

All walking	• Run a pedometer challenge (personal, team, departmental, organisational, inter-company)<br>• Run a walking challenge (teams, departments, whole-workplace, inter-workplace)<br>• Set up a fantasy footfall league<br>• Run workplace walking competitions (e.g. photos, distance, treasure hunt)
Walking to work	• Hold one off or regular walk to work breakfasts<br>• Implement a 'walk once a week' incentive scheme<br>• Hire a fitness instructor to lead a 'commute boot camp' where staff do higher-level fitness training on their walk to or from work<br>• Set up deals with local businesses e.g. money off schemes for those who walk to work

Long-term activities:

All walking	• Display motivational posters within the workplace to promote walking<br>• Set up a walking buddy / bring a friend scheme<br>• Encourage employees to keep online walking diaries
Walking to work	• Run walking buses on popular routes (to get people to public transport late at night)
Walking during work	• Organise lunch time walks, or other organised walks<br>• Produce maps of the local area denoting points of interest within 5, 10, 15 and 30 minutes' walk<br>• Encourage staff to hold walking meetings
Other	• Organise a regular health check for employees, e.g. during Know Your Numbers Week

Policy development:

All walking	• Develop a company policy on walking<br>• Introduce walking inductions for new recruits<br>• Encourage staff to set up a Walking Action Group (to campaign for improvements to routes around the site)<br>• Instigate a walkability audit that will make recommendations to senior staff<br>• Instigate an audit of your workplace policies from the points of view of encouraging walking, and make recommendations to senior staff
Walking to work	• Introduce a walking mileage

Environmental/structural changes:

All walking	• Provide benches for walkers<br>• Install signage around the site which directs staff to popular walking routes
Walking to work	• Install showers for commuters<br>• Provide lockers for bulky boots, brollies, etc.<br>• Ensure lighting around the workplace is adequate for those who leave or arrive late<br>• Introduce ‘crunchy’ pavements for late nights, so people can hear others approaching
