# Supplementary material for: Evaluation of the implementation of a whole-workplace walking programme using the RE-AIM framework
Source: BMC Public Health. 2017 May 18;17:466. doi: 10.1186/s12889-017-4376-7 (PMC5437663; doi:10.1186/s12889-017-4376-7)
Supplement: Supplementary file 2 — Employee characteristics at baseline and follow-up (PDF 170 kb) [file 12889_2017_4376_MOESM2_ESM.pdf]

Additional File 2 Employee characteristics at baseline and follow-up

Table A1 Employee characteristics at baseline and follow-up

**Characteristic**	**Baseline**		**Follow-up**		
	**n=1544**		**n=918**		***p***
	**n[a]**	**%**	**n[a]**	**%**	
**Gender**					
Female	932	68.3	526	65.6	0.189
**Age (years)**					
16-30	436	34.2	247	31.3	0.251
31-44	491	38.5	331	42.0	
≥45	348	27.3	210	26.6	
**Ethnicity**					
White	1252	93.1	730	91.9	0.466
**Highest educational qualification**					
University degree	327	46.5	323	40.7	0.113
Higher education/certificate	143	10.6	89	11.2	
GCE ‘A’ Level	272	20.2	192	24.2	
GCSE Grades A to C	251	18.6	150	18.9	
No formal qualification	18	1.3	15	1.9	
**Distance live from work**					
≤2 miles	308	21.3	155	17.5	**0.010**
2.1-5 miles	413	28.6	249	28.1	
5.1-10 miles	333	23.0	254	28.7	
>10 miles	391	27.1	227	25.6	
**Occupation**					
Senior or Middle Manager	232	16.9	150	18.5	0.079
Professional occupation	347	25.3	167	20.6	
Clerical	751	54.9	473	58.3	
Technical or manual	39	2.8	21	2.6	
**Working hours**					
Full-time	1051	76.9	637	78.8	0.305
Part-time	315	23.1	171	21.2	
**Work-related physical activity**					
Sitting occupation	1368	91.6	813	90.8	0.269
**Physical activity levels**					
Meeting current recommendations[b]	317	23.2	181	22.6	0.754

[a] Numbers do not sum up to total due to missing responses.
[b]Assessed using a single item measure of physical activity [21]
